# Integrating food is medicine and regenerative agriculture for planetary health

**DOI:** 10.3389/fnut.2024.1508530

**Published:** 2024-12-12

**Authors:** Marcia S. Rahman, Olivia Y. Wu, Kyra Battaglia, Nicole Tichenor Blackstone, Christina D. Economos, Dariush Mozaffarian

**Affiliations:** Gerald J. and Dorothy R. Friedman School of Nutrition Science and Policy, Tufts University, Boston, MA, United States

**Keywords:** food is medicine, regenerative agriculture, sustainable food system, planetary health, nutrition intervention, produce prescription program, medically tailored meals

## Abstract

The urgent need to address both human and environmental health crises has brought attention to the role of food systems in driving climate change, biodiversity loss, and diet-related diseases. This paper explores the intersection of Food is Medicine (FIM) and regenerative agriculture (RA) as an emerging approach with the potential to help address the interconnected challenges of human and ecological health within healthcare and food systems. FIM programs, such as produce prescriptions and medically tailored meals, aim to improve health outcomes by increasing access to nutritious foods and promoting nutrition equity. RA, focusing on soil health, biodiversity, and reduced reliance on synthetic inputs, offers more sustainable agricultural practices that can align with FIM goals. This paper highlights key opportunities, recent policy developments, and evidence gaps, calling for concerted efforts to clearly define RA practices and foster collaboration between community, healthcare, agriculture, and policy stakeholders. Strengthening these interconnections could lead to more resilient food systems and improved health outcomes at both individual and population levels.

## Introduction

Planetary health describes the inextricable link between the biophysical, sociopolitical, and economic well-being of human societies and the earth’s natural systems (1). Advancing planetary health is challenging due to profound shifts in agricultural practices, global supply chains, meeting the needs of growing populations and dietary patterns over recent decades (2). Currently, food systems are responsible for over 30% of global greenhouse gas emissions, 50% of ocean eutrophication, 70% of freshwater use, 90% of tropical deforestation, and unprecedented rates of topsoil and biodiversity loss (2, 3). Simultaneously, many food systems contribute to nutrient-poor and excessively processed dietary patterns, contributing to both undernutrition and diet-related chronic diseases (2).

The extensive impact of food systems on planetary health highlights the urgent need to transform how food is produced, distributed, and consumed. Achieving sustainable food systems will require multisectoral collaboration across food supply chains. For instance, food producers must be supported in adopting agricultural practices that improve climate and ecosystem outcomes. Equally, consumers need equitable access to nutritious foods that support well-being, disease prevention, and treatment.

In this paper, we explore the growing connection between Food is Medicine (FIM) and regenerative agriculture (RA), two movements reshaping food systems through complementary, multilevel actions across the food supply chain. We discuss the role of produce prescription, medically tailored grocery (MTG), and medically tailored meal (MTM) programs, which provide patients with fresh produce and other healthy foods or meals through prescriptions issued by healthcare providers and organizations. While our primary focus is on regenerative agriculture, we also examine organic agriculture, highlighting key similarities and differences between the two. By examining current definitions and showcasing real-world examples of FIM and RA integration, we outline the potential co-benefits of aligning these approaches. We also identify uncertainties that are limiting progress and opportunities to facilitate this research, implementation, and partnerships to address these challenges. Engagement from policymakers, healthcare practitioners, farmers, community organizations, patients, and researchers will be crucial in advancing the integration of FIM and RA to help promote a healthier planet and population.

## Food is medicine overview

Food is Medicine (FIM) is a healthcare strategy aimed at improving health outcomes and promoting health equity by incorporating nutritious food into disease prevention, management, and treatment (4, 5). The initiatives within FIM prioritize the inclusion of nutrient-rich foods to support healthier dietary intake and habits (6). FIM encompasses a range of interventions with varying levels of intensity, tailored to the complexity and needs of patients, from more intensive therapies like MTMs designed for patients with complex medical conditions and high healthcare usage to population-level policies and programs aimed at reaching general populations (4). Evidence supports the benefits of Food is Medicine interventions in improving food security, diet quality, health outcomes, financial strain, and mental health (4, 6). In this present review, we focus on FIM programs integrated into healthcare settings like medically tailored produce, groceries, and meals, as opposed to more population-level healthy food programs and policies.

## Organic and regenerative agriculture

### Organic agriculture

The U.S. Department of Agriculture’s National Organic Program defines organic agriculture as “the application of a set of cultural, biological, and mechanical practices that support the cycling of on farm resources, promote ecological balance, and conserve biodiversity (7).” To be considered organically grown, agricultural producers need to comply with a uniform set of standards in their production practices. These standards prohibit the use of synthetic fertilizers, pesticides, and genetically modified products (8). While not required, many organic farmers implement sustainable production practices to naturally improve the nutrient content of soil and to manage pests. These practices include the use of manure, reduced tillage, as well as crop rotation and diversification. In the United States, producers who market their produce as organic must undergo USDA organic certification, which can be time and cost-prohibitive for small or limited-resource operations (9–12). From an environmental perspective, organic systems often have lower yields than conventional, with a marginal positive contribution to reduced greenhouse gas emissions (13). Globally, on average, organic systems use less energy and have positive impacts on ecosystems by improving or preserving biodiversity, soil health, and stability, as well as water quality (13, 14). Limited evidence suggests that some products produced using organic practices have a higher density of individual nutrients (e.g., vitamin C and polyphenol levels) than their conventional counterparts, as well as lower pesticide residues (15). More research is needed to understand if consuming organic food has increased benefits to human health.

### Regenerative agriculture

The concept of RA gained prominence in the 1970s and has been championed by several groups including the Rodale Institute, a pioneering organization in sustainable farming practices. RA focuses on building and improving soil health, utilizing grazing livestock, eliminating synthetic inputs, and enhancing biodiversity, all with goals of creating economically and biologically stable agricultural systems (16, 17). As defined by Robert Rodale, RA aims to increase land and soil productivity while minimizing environmental impacts and reducing reliance on non-renewable resources by emphasizing the interconnectedness of all elements within a farming system, including the farmer (16). It is distinct from organic farming in that it does not necessarily employ organic practices—although there is often the assumption that it does. While RA and local food systems may sometimes overlap, they are not inherently linked. For example, one could support local farms without RA practices, or purchase RA products from non-local regions. While the interest in RA has gained recent attention, the practices that characterize regenerative farming today have been used within Indigenous systems for millennia (18). Practices that are foundational to RA including, but not limited to tillage reduction, crop rotation, intercropping, rotational grazing, cover cropping, and agroforestry, all emerged in pre-colonial Indigenous communities (18).

Despite these foundational principles, the definition and use of the term RA remain fluid, varying based on outcomes, processes, or a combination of the two (19). This variability may be attributed to geography and contextual factors, requiring place-based specificity (17). In the absence of a single accepted definition, variability can also be attributed to marketing efforts by heterogeneous producers to declare their practices as “regenerative.” In one review, the most mentioned RA practices emphasized minimal external inputs, such as synthetic fertilizers and pesticides, and the utility of on-farm inputs like compost and manure from grazing animals (19). The most frequently cited outcome of RA was improved soil health, followed by enhanced biodiversity, and better water retention. In terms of human health, limited evidence suggests crops grown with regenerative agricultural practices that combined no-till, cover crops, and crop rotations may also have higher nutrient densities, including increased levels of vitamins, minerals, and beneficial phytochemicals (20). Thus, enthusiasm is growing about the potential of RA to build resilient agricultural systems that can adapt to climate change, reduce environmental impacts, and ensure continued production of nutritious foods (21).

At the same time, the promise and hope may be outpacing the evidence (22). For example, evidence that RA can meaningfully contribute to carbon soil sequestration is mixed (23). Additional concerns surrounding soil carbon measurements include accurately measuring natural carbon flux throughout the agricultural system to quantify net carbon emissions from agricultural production strategies (24). Some agricultural producers are combining regenerative and organic practices on their farms and may seek certification through the Regenerative Organic Alliance to become Regenerative Organic Certified (25). While recognizing adherence to these practices can be important, ensuring that standardized regenerative certification is accessible, particularly for marginalized, diversified, and/or limited-resource producers, is also critical.

Regen10, a collaborative global initiative working to support an equitable and regenerative food systems transition, is developing a farmer-centric, outcomes-based framework focused on environmental, economic, and socio-cultural outcomes at both farm and landscape levels (26). Regen10’s approach includes dialogue with stakeholders, on-the-ground trials, and consultations to incorporate the needs of farmers, landscape stewards, and Indigenous communities. This framework aims to enable the collection of primary data, offering insights into how different agricultural practices impact diverse outcomes. Introduced at COP28, the “zero draft” framework will undergo testing throughout 2024 to refine its indicators and metrics, with the goal of aligning regenerative practices with broader global objectives such as the Paris Agreement and the UN Sustainable Development Goals. This framework could help fill key evidence gaps in regenerative agriculture.

## Interlinking food is medicine with regenerative agriculture

Food procurement within FIM programs represents an opportunity to advance planetary health by sourcing whole foods (produce prescriptions and medically tailored groceries) or ingredients (MTM) produced using sustainable agricultural practices, such as those used in certified organic or regenerative systems. Certified organic products and ingredients are widely available at a variety of outlets from large supercenters to farmers markets (27). Regeneratively produced products are more difficult to define and source given the absence of any federal or other widely accepted certification system. Based on our interactions with FIM programs, most programs who are sourcing regenerative products are doing so through local and regional food systems, e.g., by partnering directly with local farms. In so doing, these and other FIM programs who prioritize local sourcing are aligned with the USDA’s focus on more resilient local and regional food systems and access to healthy and nutritious food in all communities (28). Such programs demonstrate how federal funding can foster collaboration between healthcare and agriculture, which can help successfully advance FIM programs (6). The 2018 Farm Bill allocated $250 million for fruit and vegetable financial incentives, with $25 million specifically earmarked for produce prescription programs through the Gus Schumacher Nutrition Incentive (GusNip) program to improve dietary health by increasing fruit and vegetable intake, reducing food insecurity, and minimizing healthcare utilization and related costs (29). Since 2019, GusNip has provided over $270 million in funding to 197 projects (29). An additional $48 million of funding from the USDA American Rescue Plan Act further supported these initiatives in 2022 (28, 30).

As FIM programs gain momentum nationally, there is growing interest in how their sourcing strategies could also yield benefits for planetary health. This shift in focus is reflected in recent policy and analytical frameworks, such as the U.S. Department of Health and Human Services (HHS) framework of FIM indicators for potential use by healthcare systems and FIM providers. One of the four domains focuses on Food Production and Sourcing Effect, with indicators that include increased sourcing of regional food for FIM, strengthened local food economies, increased support for regional food producers, and local food systems transformation and expansion (31). In September 2024, Rep. Barbara Lee introduced a bill in Congress to support the establishment, implementation, and expansion of Food as Medicine (FIM) programs (32). The Food as Medicine Waiver Grant Program would fund proposals that aim to reduce nutrition-related chronic conditions, address food insecurity, and improve health outcomes through medically supportive food interventions, giving priority to organizations or entities that provide locally or regionally sourced foods that are grown or working to transition to a covered method of production, defined as regeneratively produced, organically produced or both. The bill further defines regeneratively produced as “an integrated approach to farming and ranching rooted in the principles of soil health leading to improved target outcomes” and provides specific examples such as building soil health and restoring and maintaining water resources. Additionally, this bill would give funding priority to proposals that provide technical assistance and infrastructure support to producers using these covered methods (32). Given the urgent need for all systems, including healthcare, to address climate change and planetary health, the inclusion of sustainable practices in FIM programs is relevant and timely. This emphasis on sustainable sourcing in FIM programs has the potential to not only support community health outcomes but also mitigate the environmental impacts of food production through the agricultural practices employed (33).

### Public awareness and engagement in FIM and RA

As policy efforts seek to advance sustainable food sourcing in FIM programs, the role of consumer and patient involvement becomes increasingly important. While comprehensive data on patient and consumer awareness of FIM and RA practices are limited, addressing potential uncertainty and building strong community engagement is relevant to the success and sustainability of these initiatives. Public interest and buy-in related to food sourcing and production practices could act as drivers for producer implementation and policy changes. Academic–community partnerships, such as Community Action Boards (CABs) and Food Policy Councils (FPCs), at local, state, and regional levels, can help identify potential needs and inform strategies for effective community awareness campaigns and policy engagement (34, 35). Additionally, healthcare systems not only provide patient care but also act as training grounds, which could play a key role in educating patients and setting up future professionals for careers that support FIM and sustainable agricultural practices. At the same time, while consumer awareness may help drive demand and public support for FIM, it may not be critical for FIM and RA effectiveness. Many healthcare therapies are successfully implemented based on their effectiveness rather than direct public demand.

### Incorporating local food systems and RA into FIM

**Figure 1:**
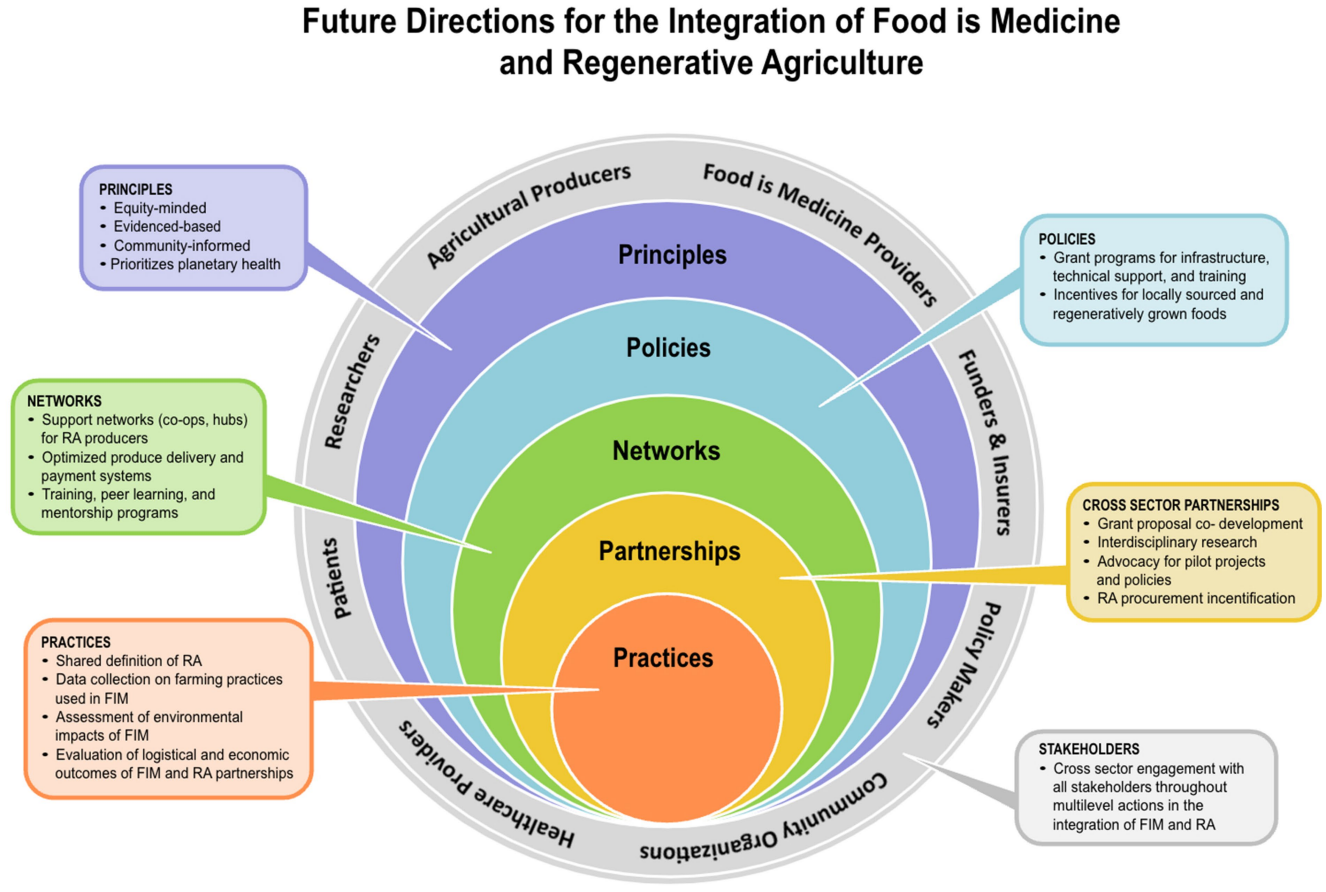
Future directions for the integration of food is medicine and regenerative agriculture.

Several real-world examples highlight both the progress made and the gaps that need to be addressed in merging FIM with specific sourcing criterion (Figure 1).

#### Recipe4Health

Recipe4Health (R4H), is an innovative FIM program in Alameda County, California that addresses both nutrition insecurity and economic barriers to food access, and focuses on environmentally sustainable, local procurement practices that aim to advance equity (36). Currently, R4H serves eligible participants who are food insecure and/or have cardiometabolic risk factors such as obesity, type 2 diabetes, or hypertension. Recipe4Health sources produce from Dig Deep Farms, a regenerative, organic farm led by local, Black, Indigenous, and People of Color (BIPOC) farmers who employ formerly incarcerated individuals. Produce boxes are delivered weekly for 16-weeks and contain predominantly vegetables and some fruits as part of the Food Farmacy. Thus far, R4H participants have significantly improved their fruit and vegetable consumption, rates of food insecurity have improved, physical activity levels have increased, and there have been improvements in chronic disease biomarkers such as HbA1c and non-HDL cholesterol when compared to a propensity matched control group over the course of 12 months (37). There is ongoing research to assess the environmental impacts of the R4H program.

#### Foodshed

Foodshed is a farmer-owned and operated cooperative in San Diego, CA that aims to address food insecurity, support local small-scale farmers, and build climate resilience, with a focus on RA practices since 2018 (38). Recognizing the challenges small RA farmers faced in scaling up and maintaining economic viability, Foodshed was created to pool resources and reduce risk across multiple farms, helping farmers access larger markets and distribute their produce efficiently. Through a collaborative crop planning model, they work closely with a core group of farmers to ensure a continuous supply of varied produce that meets market needs. The cooperative purchases fresh produce from 60 small-scale farmers prioritizing BIPOC-owned farms and those that employ RA practices (38). Foodshed’s ability to support RA and scale its operations was significantly boosted by federal funding from the American Rescue Plan Act, alongside a $5 million grant from the USDA’s Partnership for Climate Smart Commodities that enabled the cooperative to offer financial incentives for farmers to implement RA practices while also paying them a premium to supply food to historically underserved communities. Foodshed currently supplies the San Diego Foodbank with local produce on a weekly basis and is building toward expanding into FIM to support produce prescription programs in healthcare. This example highlights the potential value of a cooperative model for linking small-scale farmers with larger organizations to access and support new markets, and the important role of government grants for growth and scaling.

#### Community servings

Community Servings is a large non-profit organization based in Jamaica Plain, Massachusetts, that provides medically tailored meals and nutrition services to critically and chronically ill individuals and their families (39). Community Servings serves nearly 4,000 clients annually, delivering over 875,000 made-from-scratch meals per year (40). Community Servings local food program is a critical part of their mission and one of the ways they contribute to building a local, sustainable, community driven food economy. Through partnerships with local farms, purveyors, and food rescue organizations, they receive over 50,000 pounds of donated produce annually, including high-quality “seconds” (imperfect produce) that might otherwise go to waste (41). By sourcing locally and implementing efficient inventory management, Community Servings not only supports local farmers but also ensures that nutritious, minimally processed foods reach those in need, reducing both waste and environmental impact (42). While currently Community Servings does not include any formal RA criteria in their sourcing, they do take farming practices into account and prioritize local farms to help support a resilient, sustainable regional food system.

#### Delta GREENS

A study is underway in the Mississippi Delta to test whether a multi-level, community-engaged intervention designed to build a sustainable food economy through Food is Medicine (FIM) programming can improve minority health outcomes and reduce health disparities (43). The Delta GREENS FIM Project is a collaboration between Tougaloo College, the Ruben V. Anderson Center for Justice, Delta Health Center, Center for Science in the Public Interest (CSPI), and Tufts University. The project directly engages with local farmers to grow fresh produce for FIM prescription boxes, emphasizing the importance of clear communication, strong community partnerships, and ongoing support to achieve its goals.

The project delivers fresh, locally grown produce to underserved populations, offering valuable insights into the complex dynamics of Food is Medicine (FIM) programs, particularly in rural settings. It addresses key challenges such as selecting crops that meet both nutritional needs and local farming capabilities and overcoming logistical barriers like transportation and storage. This study highlights essential elements for sustaining FIM initiatives, especially for marginalized producers, while underscoring the broader structural supports required to create sustainable, community-driven food systems that advance both health equity and regional food security. Though Regenerative Agriculture (RA) is not formally integrated, the project’s focus on local sourcing and community-based food systems aligns with RA principles by promoting sustainability, reducing dependency on external suppliers, and fostering resilience in the regional food economy.

FIM projects that engage with local food systems may help strengthen the connection between food production and consumption. By sourcing from local producers, these programs may contribute to building more resilient regional food systems and have the potential to increase the availability of culturally relevant foods tailored to local markets (6).

### Future directions

While the integration of FIM and RA has potential for the promotion of planetary health, key uncertainties remain to be explored (Figure 1).

#### Practices: defining RA

The lack of a widely accepted and measurable definition of RA among farmers, practitioners, and researchers can hinder fruitful collaboration and policy efforts to promote widespread adoption of RA practices, including within FIM procuring pathways. While a Congressional bill has been introduced which includes a more standardized definition of RA, it is not yet finalized, leaving uncertainty around the standardization, adoption, and implementation of RA practices. In the context of FIM programs, establishing a definition for RA that accounts for regional variations in feasibility and context is essential. Efforts like Regen10’s farmer-centric, outcomes-based framework, which integrates environmental, economic, and socio-cultural dimensions, could provide valuable insights for shaping standardized yet adaptable definitions of RA. Any final definition should also consider previously silenced Indigenous epistemologies around the world and be rooted in values of reciprocity, respect, collective well-being, knowledge co-creation, and (re)localization (18). While flexibility and regional variations are important for farmers’ accessibility, some level of standardization is needed to align stakeholder efforts and foster broader policy support for RA (19, 33). Collecting data on farming practices, assessing environmental impacts, and evaluating logistical and economic outcomes are critical steps in supporting the scalability and standardization of RA. These efforts ensure that RA aligns with regional contexts and helps build evidence for its broader adoption within FIM programs.

#### Cross-sector partnerships

Cross-sector partnerships are crucial to the success of FIM programs that prioritize RA (44, 45). In addition to healthcare systems, academic institutions, and payers, government agencies, foundations, NGOs, and community-based organizations also play key roles. Together, these entities can drive the shift toward sustainable, health-focused interventions that improve patient outcomes and build the necessary support for environmentally responsible food sourcing.

Healthcare systems can establish long-term forward contracts with RA producers for FIM programs to ensure a reliable supply. Additionally, healthcare systems can align other procurement policies with value-based purchasing models that incentivize the sourcing of RA products, thus accelerating demand. Academic scientists should conduct interdisciplinary research that evaluates the potential dual benefits of RA-produced foods on patient health outcomes and environmental sustainability. Furthermore, by collaborating with farmers and community partners, academic researchers can co-develop grant proposals that study both health needs and ecological goals, helping to ensure that research outcomes translate into practical solutions that benefit the communities involved. Payers can further advance this field by financially supporting FIM programs that source from RA farms, funding pilot studies on their effectiveness, and advocating for policy reforms to consider RA foods in Medicaid and Medicare reimbursement frameworks.

#### Networks and education

Given the general small scale of RA farms at this time, building a cooperative network or food hub model can be crucial for small RA farms to support FIM programs and maintain a consistent supply of produce, especially when faced with challenges like crop failures. As highlighted in the Foodshed case study, cooperative models can allow for resource sharing and provide mutual support among farms, ensuring continuity in food production. However, these networks must also be paired with efficient distribution and payment systems to ensure timely and sustainable delivery of produce to patients in healthcare systems.

Many producers aiming to transition to RA practices also need access to training and resources to implement these strategies effectively. Programs like the USDA’s National Organic Initiative through the Environmental Quality Incentives Program (EQIP) provides assistance to producers transitioning to organic farming by helping them develop region-specific approaches for implementing approved practices (46). A similar program tailored for RA could help overcome the technical assistance barriers and lapse in production and revenue, producers may face when transitioning. The proposed Farmer to Farmer Education Act, part of the 2023 Farm Bill, aims to build networks that connect farmers with mentors and group learning experiences, supporting their efforts to adopt consistent, science-based, and site-specific conservation practices for long-term success (47). Additional resources are needed as farmers transition to RA practices.

#### Policy

Policy can play a crucial role in fostering and building both the infrastructure and the evidence base for interlinkages between FIM and RA. Existing USDA grant programs, such as the Local Food Promotion Program (LFPP) and the Regional Food Business Centers initiative, offer potential avenues for expanding support to FIM and RA projects. These programs could be tailored to include specific funding for infrastructure that supports FIM initiatives, including investments in cold storage, transportation, and aggregation hubs. Such enhancements would enable small and mid-sized farms using RA practices to distribute their products more efficiently to healthcare organizations. Additionally, these grants could provide technical assistance to help farmers navigate healthcare partnerships and meet regulatory requirements, further strengthening the connection between FIM and RA.

The Centers for Medicare and Medicaid Services (CMS) could also help drive the adoption of locally sourced and sustainably produced food in FIM programs by incorporating specific metrics and health outcome goals into Medicare and Medicaid reimbursement requirements to incentivize healthcare providers to source regeneratively grown food. This would not only support patient outcomes but also support farmers using or transitioning to regenerative practices by providing a market for their products. Finally, USDA and NIH could partner on research to further explore the food and health benefits of regeneratively grown food. A collaborative research agenda could focus on long-term studies that assess the impacts of RA-sourced food on chronic diseases, nutrient density, and overall patient well-being and satisfaction. Such research would provide the evidence needed to solidify the role of RA within FIM programs, making it easier for policymakers, healthcare providers, and farmers to work together to build a more sustainable and equitable food system.

Encouraging the adoption of RA practices through financial incentives, ecological training, and technical support within government-funded programs can accelerate their widespread use while promoting both health and environmental stewardship (6, 48, 49). This approach also levels the playing field, particularly for small or mission-driven organizations, such as non-profits, and novel healthcare programs prioritizing planetary health, such as FIM, that might face competitive disadvantages due to limitations in infrastructure, scale, or funding. Federal policies promoting RA will require a standardized definition and metrics of RA practices.

#### Principles

The intersection of FIM and RA requires a focus on planetary health, placing environmental sustainability as central to healthcare and food sourcing strategies. Approaches should emphasize equity, making nutritious, sustainably grown food accessible to vulnerable and underserved communities. Policies and practices must be evidence-based, supported by research that measures both health and environmental outcomes. Additionally, community engagement is critical, ensuring that interventions are responsive to local conditions and grounded in the values and priorities of the communities they serve.

## Conclusion

To protect both human and environmental health, we must reorient our food systems to be nourishing, equitable, and sustainable. The shift can be supported locally, with consumers, communities, and institutions driving sustainable practices. Local actions can model broader, systemic change needed to meet global health and sustainability targets. However, the adoption of healthy diets from sustainable food systems will require more than individual actions—it will call for a collective restructuring of how we fund, produce, distribute, and consume food, with an emphasis on reducing environmental impact while ensuring food equity for all (50). As one potential solution, an intersection between FIM and RA could help advance these goals. Achieving this vision will require new research, cross-sector partnerships, networks, and policies that prioritize the intersections and uncertainties that interlink our food with human and planetary health.

## Data Availability

The original contributions presented in the study are included in the article/supplementary material, further inquiries can be directed to the corresponding author.
